# Development of Pulsating Twin Jets Mechanism for Mixing Flow Heat Transfer Analysis

**DOI:** 10.1155/2014/767614

**Published:** 2014-02-02

**Authors:** Ali Ahmed Gitan, Rozli Zulkifli, Shahrir Abdullah, Kamaruzzaman Sopian

**Affiliations:** ^1^Department of Mechanical and Materials Engineering, Faculty of Engineering and Built Environment, Universiti Kebangsaan Malaysia (UKM), 43600 Bangi, Selangor, Malaysia; ^2^Department of Mechanical Engineering, College of Engineering, University of Tikrit, Tikrit, Iraq

## Abstract

Pulsating twin jets mechanism (PTJM) was developed in the present work to study the effect of pulsating twin jets mixing region on the enhancement of heat transfer. Controllable characteristics twin pulsed jets were the main objective of our design. The variable nozzle-nozzle distance was considered to study the effect of two jets interaction at the mixing region. Also, the phase change between the frequencies of twin jets was taken into account to develop PTJM. All of these factors in addition to the ability of producing high velocity pulsed jet led to more appropriate design for a comprehensive study of multijet impingement heat transfer problems. The performance of PTJM was verified by measuring the pulse profile at frequency of 20 Hz, where equal velocity peak of around 64 m/s for both jets was obtained. Moreover, the jet velocity profile at different pulsation frequencies was tested to verify system performance, so the results revealed reasonable velocity profile configuration. Furthermore, the effect of pulsation frequency on surface temperature of flat hot plate in the midpoint between twin jets was studied experimentally. Noticeable enhancement in heat transfer was obtained with the increasing of pulsation frequency.

## 1. Introduction

The main idea of producing pulsed air jet is the frequent opening and closing of the nozzle exit which are based on the required pulse frequency. Basically, the common ways to produce pulsed air jet include using the solenoid valve system, rotating valves and acoustic speakers [1–3]. The specifications of these three types will be illustrated briefly just to determine our selection depending on the desired features.

The solenoid valve system was designed to be controlled by electrical current which produces magnetic force governing the opening and closing of the solenoid plunger and then the valve. This operation technique and valve design characterized the valve with some features. One of these features is the limitation of inlet fluid pressure under which the valve works. In addition, the low opening and closing frequency of the valve may limit the application field of this type of valves especially in producing pulsed jet flow. These are some relevant characteristics that may lead us to consider the other ways of pulsed jet flow production.

The acoustic speakers can also be used to generate pulsed jet flow with some limited conditions by directing it on a steady flow; thus an excitation of the flow results. One of these conditions is that the flow mostly should be of low velocity. This condition cannot serve our work requirement of high Reynolds number for a more effective heat transfer enhancement.

According to the above, it can be concluded that the issue of which mechanism has to be used is determined based on the research objectives. These objectives can be understood by considering the desired limits of pulsed jet mechanism operation in detail. Therefore, reviewing published researches provides further information and reveals significant findings regarding the factors to be determined in order to achieve the aims of the study.

## 2. Review of the Literature

There are many ways discussed in the previous studies of how to produce a pulsed jet or excite a steady jet. First of all, generation of pulsed jet was investigated by using acoustic excitation [3]. Also, production of pulsation in flow was considered by using synthetic jets [4]. Moreover, an interrupted flow was investigated by using rotational valve [2]. Furthermore, oscillated jet was studied by improving a self-oscillating impingement jet [5]. In brief, these are some methods mentioned in the published literatures.

One of these ways is the acoustic excitation which is studied by [3] who studied experimentally the effect of acoustic excitation on the flow and heat transfer characteristics of a jet impingement directing on a plate. Two methods of acoustic excitation based on actuator position were investigated in this work. The effect of excitation level and nozzle-to-plate distance on heat transfer were discussed. The local heat transfer on the impingement surface, velocity, and turbulence intensity were measured. The forcing Strouhal numbers (St) are 1.2, 2.4, 3.0, and 4.0 and the excitation level varies from 80 to 100 dB. The effect of excitation showed that with 1.2 Strouhal numbers low heat transfer rates result, while for 2.4 St high heat transfer levels are obtained. Both excitation methods revealed similar heat transfer characteristics although there are differences between them.

The solenoid valve was used also in [1] in order to develop an effective dry surface cleaning method for removal of fine particles. Resuspension experiments were carried out with monodisperse PSL particles and wax particles with diameter between 0.25 and 1.1 *μ*m on silicon wafer and glass plate. The results showed that the process of particles deposition on the surface affects slightly the removal efficiency and that consecutive pulsed air jet is effective in the removal of fine particles.

Reference [3] excited an axisymmetric air jet using four synthetic jets distributed around the circumference of the primary nozzle with helical and bifurcating modes. The main purpose was to investigate the influence of the actuation on the impingement heat transfer at the Reynolds numbers 1600 and 5000. The more important effects were obtained at the Strouhal numbers 0.14–0.32 at the ratio of the control to primary jet momentum rates between 0.24 and 2.4%. At small nozzle-to-wall spacing *H*/*D* = 2 the heat transfer was enhanced by 40% in the stagnation point due to excitation effect. Under moderate nozzle-to-wall spacing *H*/*D* = 6, Nusselt number distribution was shown to be more uniform due to excitation.

Reference [2] used rotational ball valve to produce pulsed jet in order to investigate the effect of flow pulsations on the local heat transfer characteristics of a planar air jet. The ball valve was driven by variable speed DC electric motor. A nozzle-to-plate distance between 0 and 10 of nozzle width was considered with pulsation frequencies ranging from 0 to 80 Hz and Strouhal numbers less than 0.106. Steady and pulsating jets are tested at jet Reynolds numbers of 1000, 5500 and 11000 with pulse amplitude at the nozzle exit ranging from 0 to 50% of the mean flow velocity. Heat transfer was enhanced up to 12% near the mid-line of nozzle because of renewal effects and up to 80% at distances downstream due to the increase of turbulence levels.

Reference [5] shows that a self-oscillating-impinging-jet configuration is extremely beneficial in enhancing the heat removal performance of a conventional (stationary) impinging jet. Moreover, the inherent sweeping motion resulting from the oscillating coolant jet causes significant enhancement for area coverage of the impingement region in addition to the Nusselt number measured at stagnation line. Nusselt number is enhanced at stagnation line by 70% when the oscillating jet has characteristics of *Re* = 14,000 and *x*/*d* = 24 hole-to-plate distance. The current findings suggest that it may be possible to implement the present self-oscillating-impinging-jet concept in future gas turbine cooling systems, rotating disks, glass tempering/quenching, electronic equipment cooling, aircraft deicing, combustors, and heat exchangers.

As a result, the rotating valve principles are more suitable for a pulsed jet mechanism that fits many desirable conditions. Firstly, higher pulse jet frequency mechanism is more useful to a wide range of studies. Also, the system should withstand high Reynolds number and high pressure inlet flow to the valve. In addition, multijet system with variable jet parameters is helpful to extend the study in the field of pulsed jet flow. Several factors were considered as important criteria in the present design. One of these factors is the variable phase between the pulse frequencies that enable us to study the effect of phase change. The other factor is the jet-to-jet distance that helps us to discover the influence of jets interaction. These are the reasons why the rotational valve was selected and developed in this study which was implemented according to logical procedures as presented in the next item.

## 3. Methodology 

As mentioned in the last paragraph, the rotating valve was chosen as a base of the pulsed jet production valve design due to the competence of this type of valves. The logical thinking of the present design was done as follows:

- considering the pulsed jet mechanism proposed in [6] as a starting point of our design;
- developing this design from single jet to twin jets mechanism;
- proposing a way to make the jet-to-jet distance variable;
- considering this proposal to conform to making the pulse frequencies of jets have variable phase difference;
- adjusting the design to fit the two variable factors of jet-to-jet distance and phase difference in jets pulse frequencies;
- testing the fabricated mechanism to verify the precession of design and fabrication. This step can be achieved by conducting two flow field tests: first, testing the velocity profile of pulsating frequency; second, testing velocity profile across the jet diameter.

## 4. Modified Pulsed Jet Mechanism

The principles of pulsed jet mechanism presented in this work are inspired by rotating valve with some modifications. Basically, the idea of producing pulsed jet results from using rotating cylinder enclosed inside block. The rotating action was achieved by connection of the cylinder with electrical AC motor by a shaft. This cylinder and its block housing were bored on one line so that the air flow can enter without any blockage except that of cylinder wall when it rotates. The valve will open when the bore of rotating cylinder faces that of block housing during the rotation of cylinder and it will close when these bores are not on one line. The repeated opening and closing cause pulsed air flow and whenever the speed of rotation increases, the pulse frequency increases too. Additional design features were established to produce two pulsed jets with certain specifications.

**Figure 1:**
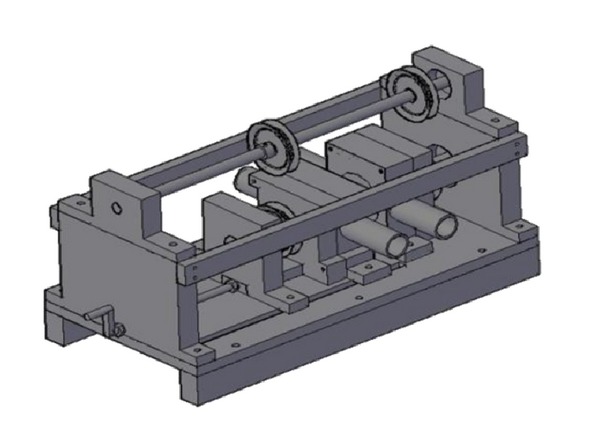
Drawing of PTJM.

**Figure 2:**
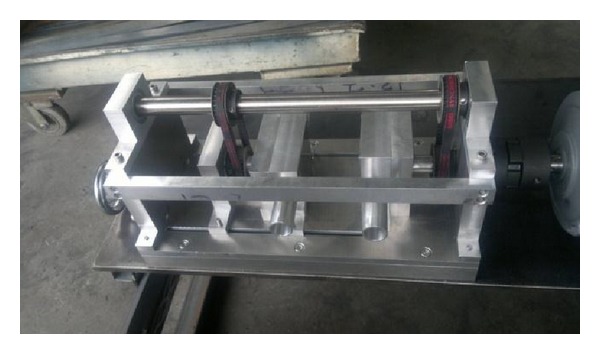
Modified PTJM.

**Figure 3:**
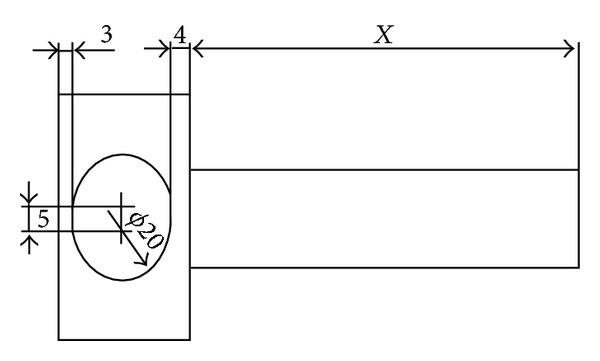
Rotating part in which its hole is based on 33% duty cycle.

Pulsed jet mechanism modification, which its drawing and its picture just after fabrication are shown in Figures 1 and 2 respectively, was processed in three main steps taking into account the new design parameters required. First of all, the replacement of single jet by twin jets was considered and achieved by boring the rotating cylinder and housing block with two bores of 20 mm diameter instead of one bore. It is important here to mention that the duty cycle which is the ratio of pulse cycle on-time to the total cycle time was fixed at 33% for the present design based on a previous study [7] as shown in Figure 3, which shows the hole of the rotating part based on duty cycle calculations.

**Figure 4:**
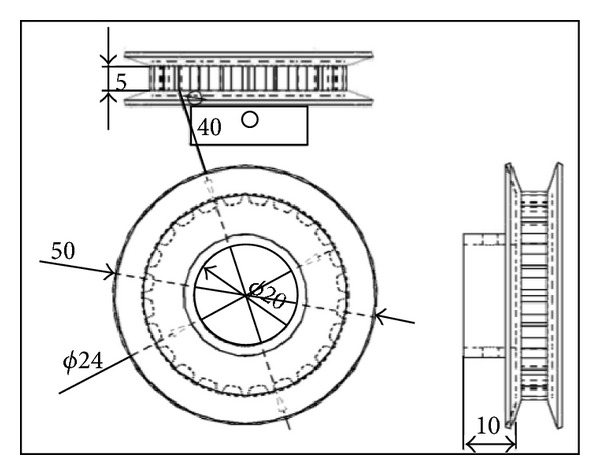
24 teeth timing belt pulley.

Secondly, in order to make the design fit with phase change of pulse frequency between the two jets, the two-bored cylinder was separated into two rotational parts as shown in Figure 3, where both rotational parts coincide with all dimensions except for the length of the shaft (*x*). Thus, it is easy to set the desired phase angle between the pulses frequencies of the two jets by rotating one of the two rotational parts manually and keeping the other fixed. The minimum phase angle pitch is 15°; therefore, 24 teeth were selected for the timing pulleys installed on shafts of the rotational parts as illustrated in Figure 4. Those pulleys are responsible for transmission of rotational motion from the shaft of the right rotational part that is driven by AC motor to the upper shaft and then to the shaft of left rotational part.

**Figure 5:**
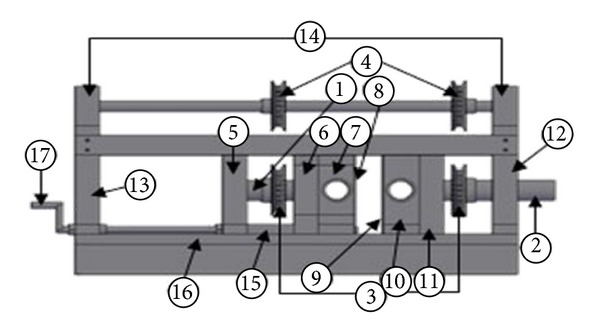
Components of the mechanism.

Last but not least, a novel design idea of the jet-to-jet distance control mechanism was designed to fulfill the nearest vicinity of two jets of 10 mm. This specification was achieved by designing one of the two nozzle assemblies as a movable part and the other was kept fixed. As shown in Figure 5, the movable nozzle assembly consists of parts 5, 6, 7, 8, 15, 1, and one of part 3 (the timing pulleys), while the fixed nozzle assembly comprises parts 9, 10, 11, 2, and one of part 3. The timing pulleys are connected by timing belts with specifications of 2800 rpm rotational speed, less noise level, and without any probability of slipping. The components of both nozzle assemblies include bearing housing (parts 6 and 11), housing of the rotational part (parts 7 and 10), and plate cover (parts 8 and 9). The movable nozzle assembly can slide by turning the rotating handle (part 17) manually, where the sliding process was designed so that every turn of handle displaces the movable nozzle assembly by 1 mm.

## 5. Generation of Pulses

The purpose of multipulsed jet system design is to produce two pulsed jets with some features of adjustable jet-to-jet distance and variable phase difference between the frequencies of the two pulsed jets. The pulsed air jet is generated by a steady compressed air coming from high pressure air tank and entering the system through two inlet holes in the housing body. When the hole of the rotating cylinder aligns with the corresponding housing body holes, the air flows through the system. On the other hand, the system stops air discharge when the rotating cylinder hole does not align with the corresponding housing hole. This frequent air interruption generates periodic pulses of air jet in which its frequency is equivalent to twice the rotational motor speed. Briefly, a mechanical technique of rotational motion of drilled cylinder was exploited to generate pulses and this technique was developed in the current study to produce many types of jet flow as illustrated in the outcome resulting from this new design.

## 6. Twin Jets Flow Field

**Figure 6:**
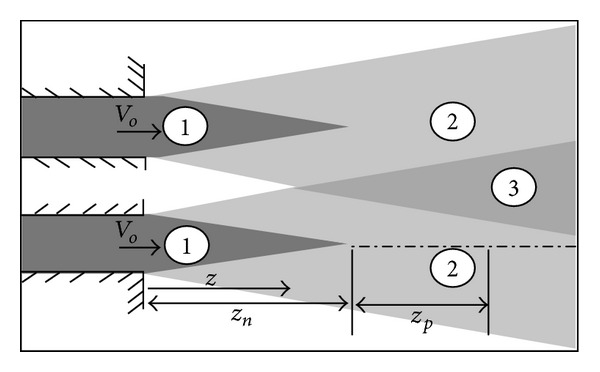
Flow field of twin free jets.

The behavior of jet flow field can be varied according to several factors. One of these factors is the physical properties of jet and ambient materials. If the jet material is freely miscible with the ambient material then a boundary layer forms as an expanded region along the axis of the jet.  Figure 6 illustrates the basic regions that can be formed in jet flow field. Region 1 which is called the core region has a constant value of velocity which is the same as nozzle flow exit velocity (*V*_*o*_). The jet fluid entrains the ambient fluid and the viscous mixing forms region 2. For twin jets flow field, the interaction of region 2 between both jets forms region 3. Basically, three regions can be recognized in jet flow field along the axis of jet. Firstly, the initial region represents the region from the nozzle exit to the intersection of the boundary layer of the core region and the axis of jet (*z* < *z*_*n*_). Secondly, in the transient region (*z*_*n*_ < *z* < *z*_*p*_) the velocity profile is not stabilized yet. Thirdly, the main region (*z* > *z*_*P*_) is the zone in which the velocity profile becomes constant. These three regions characterize the flow field of single jet. However, the flow field of twin jets still needs more analysis especially with the consideration of pulsating.

## 7. Experimental Setup and Measurements

**Figure 7:**
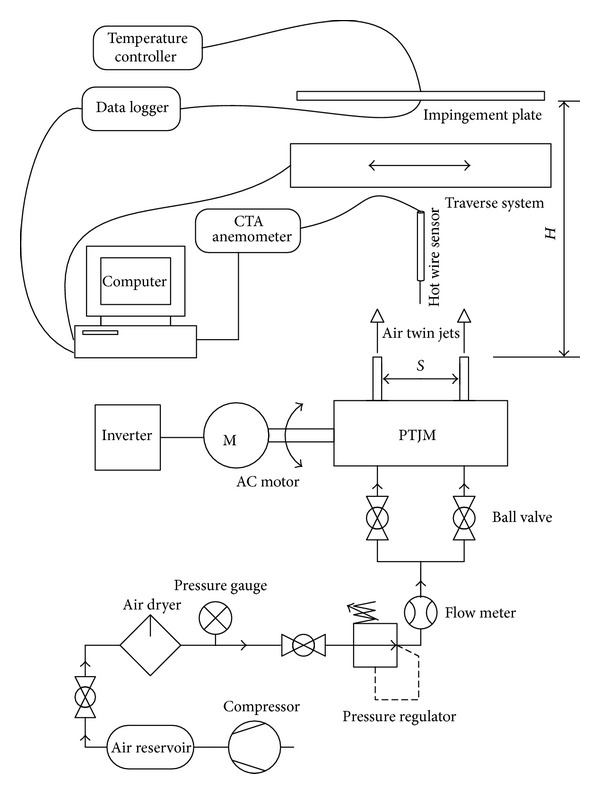
Schematic representation of the experimental setup.

A schematic of the experimental apparatus is shown in Figure 7. Compressed air was supplied from a reservoir and dehumidified by air dryer. Air pressure gauge, regulator, and flow meter were installed to control air flow. The air was divided into two branches before entering PTJM. AC electric motor controlled by SJ200 Series HITACHI inverter was used to operate the mechanism. Hot wire technique with multichannel CTA anemometer from Dantec Dynamics was used to measure the velocity of air jet. An aluminum flat plate of 300 × 300 × 5 mm dimensions was used as an impingement surface. An electric flat heater controlled by temperature controller was fixed on the back of the plate. A microfoil heat flux sensor and GRAPHTEC GL820 data logger were used to measure the heat flux and surface temperature on the impingement point. The whole domain was scanned by using one-dimension traverse system.

In the present work, the aim of the measurements is to verify the design of PTJM, which was designed to fulfill the requirements of pulsating jet impingement heat transfer studies. Basically, the measurements were conducted in two fields of studies: flow field and heat transfer tests. Flow field tests consist of pulsation frequency test and velocity profile measurements for the twin jets. The pulsation frequency test was performed by measuring the pulse of jet during a specific period of time. The pulse of jet is represented by jet velocity at its center during the time. The velocity of jet was measured by using hot wire anemometry technique. Secondly, the velocity profile test was carried out by installing the hot wire sensor in the traverse system to scan the domain in direction cross to the jet direction along the horizontal center line of both jets covering the whole region of twin jets effect. An increment of 2 mm increment was set for velocity measuring position across the flow field. On the other hand, the heat transfer test consists of measuring surface temperature of hot flat plate impinged by twin pulse jets and heat flux at different levels of five factors which are pulsation frequencies, nozzle-to-nozzle distance, nozzle-to-plate distance, and phase angle. These five factors were investigated experimentally by using design of experiment (DOE) approach due to the huge number of experiments required if one factor at a time (OFAT) method is used [8]. The response surface method (RSM) was selected to optimize the heat transfer rate. The measured point was selected in the midway between twin jets to consider the maximum interaction region behavior. The measuring of temperature was accomplished by using thin film thermocouple with data logger device. The pulsation frequency in these measurements could be controlled by using inverter device connected between the power source and the AC motor to control the signal entered to the AC motor.

## 8. Results and Discussion

The current design was carried out to achieve many types of flow patterns according to the way of operation. Firstly, the system can produce a continuous flow (steady flow) for the two nozzles when the AC motor is turned off and the holes of rotating parts are aligned with the nozzles. Secondly, keeping the same aligning of the rotating holes and turning on the AC motor provide pulsating twin jets flow with the same frequency and zero phase angle between the frequencies of both jets. Thirdly, different phase angles between the two equal frequencies can be obtained by changing the alignment of the rotating holes with each other. These three flow patterns with the ability of changing the jet-to-jet distance will give different levels of complexity in which the flow can be characterized.

**Figure 8:**
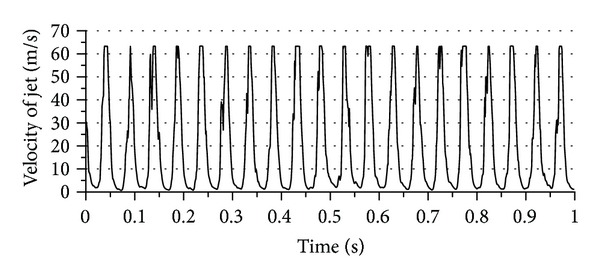
Pulse profile for jet (1) at 20 Hz pulse frequency.

**Figure 9:**
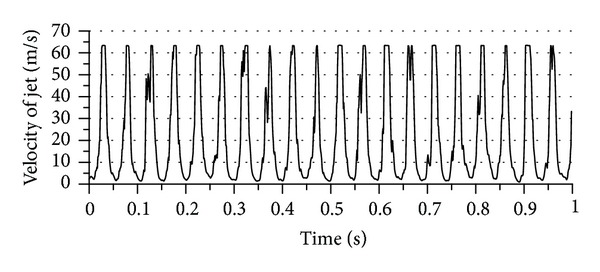
Pulse profile for jet (2) at 20 Hz pulse frequency.

**Figure 10:**
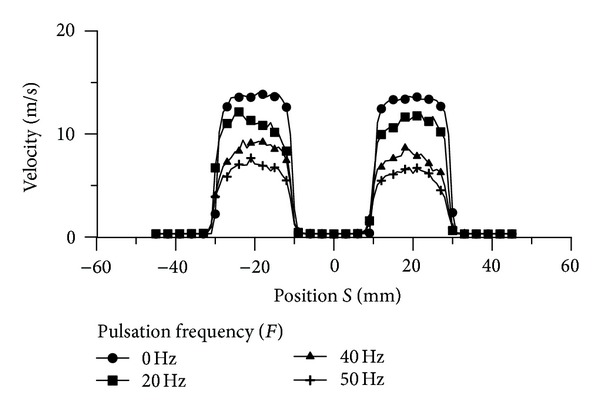
Velocity profile of the twin pulsating jets at *H* = 0 mm, *S* = 20 mm, *P* = 0.2 MPa, and *ϕ* = 0° with different frequencies.

The experimental tests were conducted for flow field and heat transfer measurements.  Figure 8 represents the pulse profile of jet (1) in the center of jet during 1 second at 20 Hz pulsation frequency, free jet-to-plate distance (*z*) of zero mm, and supplied air pressure of 0.3 MPa. It can be noticed from this figure that jet (1) issues uniform pulse jet along the specified period. Also, the cut flow point gives zero flow and this is a clear evidence for no leakage existing at cut flow point. Thus, the concept of pulsation flow was achieved. Furthermore, the number of peak points is equal to the frequency set value and this gives verification for the accuracy of frequency generation. Similarly, pulse profile during a specific period of time for jet (2) could get the same characteristics of that for jet (1) at the same conditions as shown in Figure 9. In comparison, both jets have the same value of peak velocity and this is a verification for considering both jets as twin pulse jets. The velocity profile across the jet diameter was tested as a second verification for the mechanism at free jet-to-plate distance (*z*) of zero mm, jet-to-jet distance (*S*) of 20 mm, supplied air pressure (*P*) of 0.2 MPa, and phase angle (*ϕ*) of 0° as illustrated in Figure 10. A reasonable velocity profile can be seen at pulsation frequency (*F*) of zero, where flat profile is one of the steady jet characteristics close to nozzle exit, where the core region dominates the whole jet region as mentioned earlier in this paper. Another point of view can be discussed for this figure, which is the high similarity between both jets qualitatively and quantitatively. This can be considered as a good indication for the coincidence of the twin jets. The main purpose of this novel design is to study the region of interaction between jets from two points of view: the flow field and heat transfer aspects.

**Figure 11:**
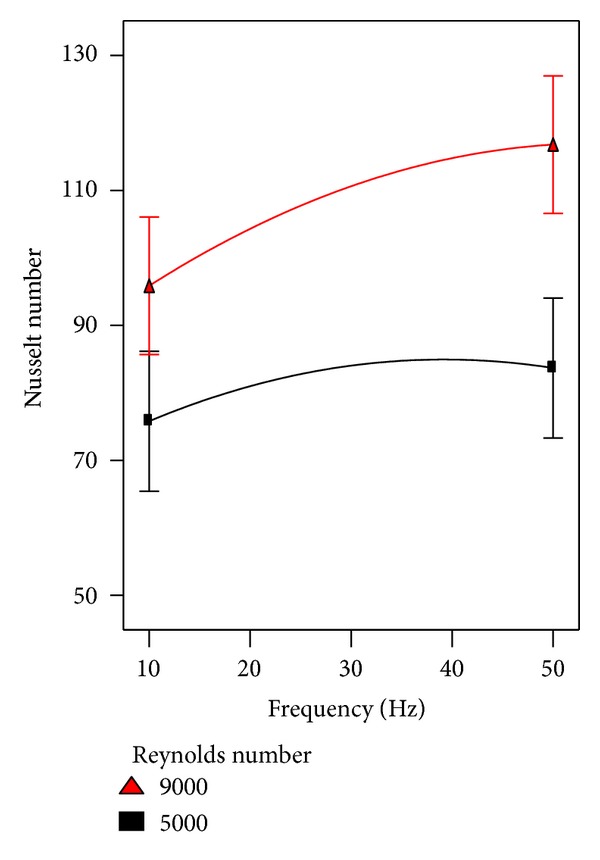
The effect of frequency involved with Reynolds number on heat transfer.

**Figure 12:**
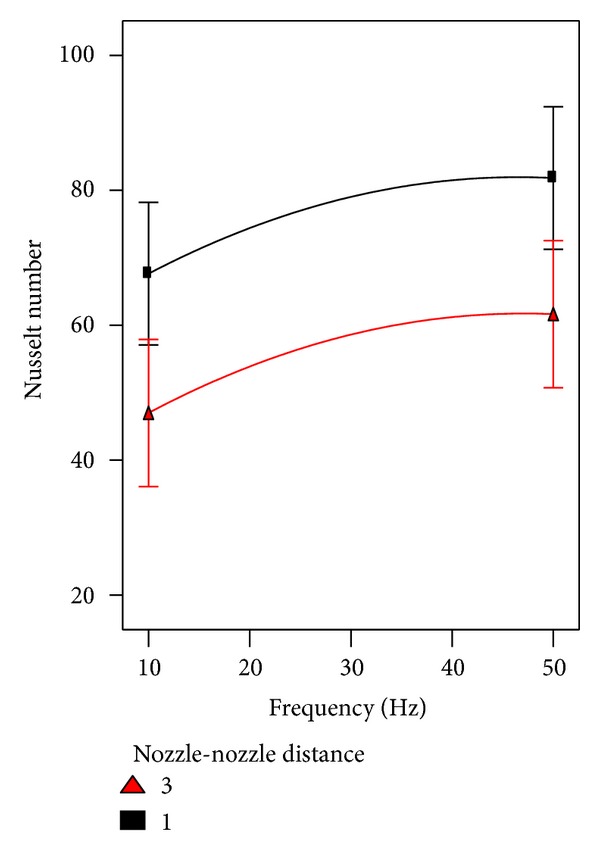
The effect of frequency involved with nozzle-nozzle distance on heat transfer.

**Figure 13:**
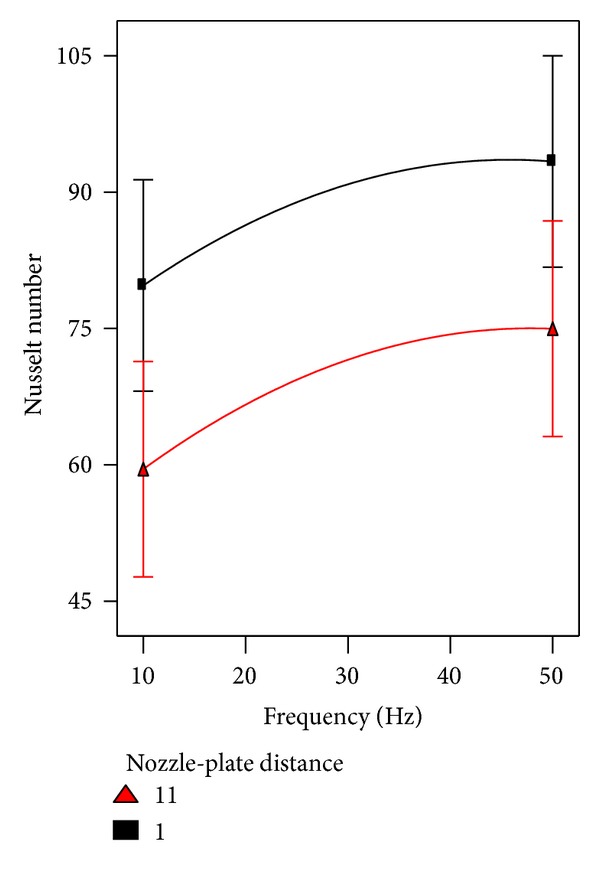
The effect of frequency involved with nozzle-plate distance on heat transfer.

Regarding the heat transfer consideration, the effect of pulsation frequency on Nusselt number was considered involving other factors. The interaction between pulsation frequency and Reynolds number and their effect on heat transfer are shown in Figure 11. Obviously, it can be seen from the figure under discussion that there is noteworthy enhancement in heat transfer at higher frequencies especially at higher Reynodls number. The interaction of pulsation frequency and nozzle-nozzle distance is illustrated in Figure 12. No interaction can be noted between both factors, where the nozzle-nozzle distance has no effect on the behavior of pulsation frequency that improved the heat transfer.  Figure 13 which represents the interaction between pulsation frequency and nozzle-plate distance can tell the same story of Figure 12.

**Figure 14:**
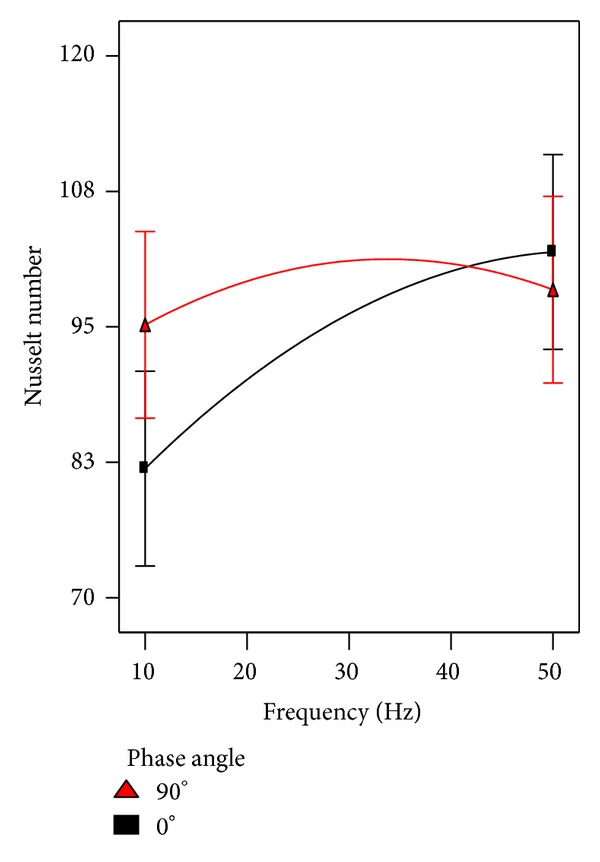
The effect of frequency involved with phase angle on heat transfer.

The phase angle and pulsation frequency interaction is presented in Figure 14. The phase angle of 90° achieved higher heat transfer rates at frequency less than 40 Hz. However, the higher Nusselt number was recorded at frequency of 50 Hz when the twin jets flow in phase.

## 9. Conclusion

In the present work, the development of pulsed jet mechanism was achieved based on the research objectives and factors considered. In the inception, a review of many pulsed jets producing systems was established. Also, the design methodology of the pulsed jet system was stated based on logical design steps. Moreover, the developed pulsed jet mechanism was described in details. Furthermore, the operation of pulsed jet device was presented to illustrate the novel design idea. In addition, the twin pulse jets mechanism was tested by different ways. Firstly, flow field tests including pulse velocity profile during a specific time and velocity profile for twin jets through their cross sections were conducted. Velocity peak of around 64 m/s for both jets was obtained by measuring velocity with time under pulsation effect and around 20 m/s by testing velocity across the diameters of steady jets. Secondly, the mechanism was tested from the heat transfer point of view by measuring the heat transfer on hot plate impinged by twin jets at midpoint between jets at five different factors. A noteworthy improvement of heat transfer was achieved with pulsating twin jets mechanism. In conclusion, a more competent and verified pulse twin jets mechanism was developed to fulfill the experimental tests requirement by considering several important factors and consequently presenting clear description of pulsed jet impingement for heat transfer problems.

## References

[1] Otani Y, Namiki N, Emi H (1995). Removal of fine particles from smooth flat surfaces by consecutive pulse air jets. *Aerosol Science and Technology*.

[2] Mladin EC, Zumbrunnen DA (1997). Local convective heat transfer to submerged pulsating jets. *International Journal of Heat and Mass Transfer*.

[3] Hwang SD, Cho HH (2003). Effects of acoustic excitation positions on heat transfer and flow in axisymmetric impinging jet: main jet excitation and shear layer excitation. *International Journal of Heat and Fluid Flow*.

[4] Trávníček Z, Nĕmcová L, Kordík J, Tesar V, Kopecký V (2012). Axisymmetric impinging jet excited by a synthetic jet system. *International Journal of Heat and Mass Transfer*.

[5] Herr F, Camci C Turbulent transport in a planar jet with self-sustained deterministic oscillations.

[6] Zulkifli R, Sopian K, Abdullah S, Takriff MS (2009). Comparison of local nusselt number for steady and pulsating circular jet at Reynolds number of 16000. *European Journal of Scientific Research*.

[7] Sailor DJ, Rohli DJ, Fu Q (1999). Effect of variable duty cycle flow pulsations on heat transfer enhancement for an impinging air jet. *International Journal of Heat and Fluid Flow*.

[8] Montgomery DC (2009). *Design and Analysis of Experiments*.

